# Intrapersonal, social-cognitive and physical environmental variables related to context-specific sitting time in adults: a one-year follow-up study

**DOI:** 10.1186/s12966-016-0354-1

**Published:** 2016-02-27

**Authors:** Cedric Busschaert, Ilse De Bourdeaudhuij, Jelle Van Cauwenberg, Greet Cardon, Katrien De Cocker

**Affiliations:** Department Movement & Sport Sciences, Ghent University, Watersportlaan 2, 9000 Ghent, Belgium; Department Public Health, Ghent University, 9000 Ghent, Belgium; Department of Human Biometry and Biomechanics, Vrije Universiteit Brussel, 1050 Brussels, Belgium; Fund for Scientific Research Flanders (FWO), Egmontstraat 5, 1000 Brussels, Belgium

**Keywords:** Adults, Correlates, Predictors, Longitudinal, Sitting time

## Abstract

**Background:**

Investigating associations between socio-ecological variables and context-specific sitting time in adults can support the development of future interventions. The purpose of the present study was to examine the cross-sectional and longitudinal relationships of intrapersonal, social-cognitive and physical environmental variables with context-specific sitting time (i.e. TV-viewing, computer use, motorized transport, and occupational sitting) in adults.

**Methods:**

In this longitudinal study, data were retrieved from a random sample of Flemish (Belgian) adults. At baseline, 301 adults (age, 43.3 ± 10.6 years) completed a questionnaire on context-specific sitting time and its potential predictors. After a 1-year follow-up period, complete data of 188 adults was available (age, 46.0 ± 10.4 years). Multiple linear regression analyses were performed for both the cross-sectional data at baseline (correlates) and the longitudinal data (predictors).

**Results:**

The cross-sectional and longitudinal analyses revealed different relationships between sitting during TV viewing, computer use, motorized transport and occupation. Generally, change in cross-sectional correlates did not cause change in context-specific sitting time in the longitudinal analyses. Social-cognitive correlates/predictors were most frequently identified, followed by intrapersonal correlates/predictors. Attitude, self-efficacy, (social) norm and modelling were found to be the most consistently related social-cognitive correlates/predictors to context-specific sitting time. Limited evidence was available for relationships between physical environmental variables and context-specific sitting time.

**Conclusions:**

The cross-sectional correlates differed from the longitudinal predictors of context-specific sitting time, highlighting the need for longitudinal research. The present study also underlined the need for family interventions to minimize context-specific sitting time, as both intrapersonal and social-cognitive variables were associated with context-specific sitting time.

## Background

Sedentary behaviour (SB – too much sitting), defined as any waking activity characterized by an energy expenditure ≤1.5 metabolic equivalents (METs) performed in a sitting or reclining posture [1], receives increasing attention in terms of chronic disease prevention [2]. Too much sitting has been identified as a risk factor, independently of physical activity (PA), for all-cause mortality, fatal and non-fatal cardiovascular disease, type 2 diabetes, metabolic syndrome and incidence rates of some types of cancers in adults [3]. Besides these physical health risks, high levels of sitting time have been shown to be related to decreased mental health [4]. Further, long periods of uninterrupted sitting should also be avoided, as breaking up sitting time may be beneficial for cardio-metabolic health and more breaks were related to lower triglycerides, waist circumference, BMI, and 2-h plasma glucose among adults [5, 6]. Despite these physical and mental health risks, adults spend a lot of time sedentary throughout the day [7]. For example, Belgian adults had high self-reported (610 min/day) and inclinometer (activPAL)-determined (561 min/day) levels of total sitting time [7]. As sitting occurs in multiple contexts [8], gathering context-specific information is warranted. In Belgian adults, the largest proportion of total sitting time was obtained from occupational sitting time (193–241 min/day), followed by the following contexts of sitting time: television (TV) time (58–90 min/day), non-occupational computer time (58 min/day) and motorized transport (44–52 min/day) [7]. Similar results have been found among British adults [7]. Given the high prevalence of sitting time, future intervention studies should aim to reduce sitting time in adulthood, especially in highly-prevalent and modifiable contexts of sitting time (i.e. screen-related behaviours in leisure time, occupational sitting time and motorized transport). Gathering information on these contexts of sitting time is necessary to inform future intervention developers. Still, in order to design effective future interventions, more knowledge is warranted on predictors of context-specific sitting time [9]. Previous research predominantly gathered information based on cross-sectional studies (i.e. correlates), however, longitudinal study designs are needed to identify causal relationships (i.e. predictors). To date, the few longitudinal studies on predictors of sitting time only focused on the contexts of TV-viewing (*n* = 4 studies), computer use (*n* = 1 study) or total sedentary time (*n* = 3 studies) in adults [10–16]. However, only focussing on predictors of screen-related sedentary behaviours (SRSBs) may not be sufficient to obtain significant changes in total sitting time, since SRSBs are just one part of total sitting time. But also, only identifying predictors of total sitting time is not enough [9], as this information is too broad to design more-effective future interventions. To our knowledge, no previous study investigated predictors of non-SRSBs (i.e. motorized transport and occupational sitting). In addition, previous research, both cross-sectional and longitudinal, mostly focused on non-modifiable socio-demographic variables related to SRSBs, occupational sitting, motorized transport or total sitting time in adults [17–20]. However, as ecological models state that multiple levels of predictors influence the different contexts of sitting time [9], it is recommended to include variables of different levels (e.g. intrapersonal, interpersonal, environmental) as potential correlates or predictors of context-specific sitting time.

So, more information is warranted on both cross-sectional (correlates) and longitudinal (predictors) context-specific variables of different ecological levels of various contexts of sitting time, as research is scarce and mostly limited to socio-demographic predictors and SRSBs. The objective of the present longitudinal study with a follow-up period of 1 year was to investigate intrapersonal, social-cognitive and physical environmental correlates and predictors of context-specific sitting times in adults. First, cross-sectional correlates (i.e. intrapersonal, social-cognitive and physical environmental variables) of Flemish adults’ context-specific sitting time at baseline (i.e. TV-viewing, computer use, motorized transport and occupational sitting) were identified (aim a). Secondly, we examined if changes in these variables from baseline to follow-up predicted changes in adults’ context-specific sitting times (aim b).

## Methods

### Subjects and procedures

In this longitudinal study on sitting time and its predictors with a follow-up period of 1 year, contact information (full name, address and date of birth) from a random sample of 1917 adult inhabitants of the city of Sint-Niklaas (Flanders, northern Dutch-speaking part of Belgium) was obtained from the city’s public service department. Sint-Niklaas is a metropolitan city with approximately 73,000 inhabitants. An information letter (background, objectives and practical information of the study) and a questionnaire were sent to the adults in April 2013. From the 1917 potential participants, 1909 adults actually received these documents (one adult had moved to another address, and seven could not be reached due to incorrect addresses). Adults were asked to send the completed questionnaire to the research office in an accompanied prepaid and preaddressed envelop. A reminder was sent to all potential participants after 3 weeks to increase the response rate. In total, 334 questionnaires were returned to the research office (response rate: 17.50 %). To be included in the study, participants had to be included in the sample drawn by the public service department, aged 25- to 60-years, able to stand and living in Sint-Niklaas. Information on the possibility to stand was obtained from one of the first questions in the questionnaire. Participants who were not able to stand should only complete the first part of the questionnaire (socio-demographics). Thirty-three adults were not included in the analyses (i.e. partner completed the questionnaire: *n* = 21; person was not capable to stand: *n* = 7; refusals to participate: *n* = 2; questionnaires received after the deadline: *n* = 2; empty questionnaire: *n* = 1). This resulted in a final sample of 301 adults at baseline (15.77 %).

At follow-up (April 2014), all baseline participants received the same questionnaire accompanied with a prepaid and preaddressed envelop and an information letter. Three hundred and one questionnaires were sent and 299 adults actually received these documents (two participants had moved to another address). In total, 190 questionnaires were returned to the research office (response rate: 63.55 %). Two participants were excluded due to incomplete questionnaires, resulting in a final sample of 188 adults participating both at baseline and follow-up.

Drop-out analyses were executed to test for possible differences between the adults who dropped out and the adults who remained in the study. Adults who dropped out were slightly younger (40.23 ± 10.38 years vs. 45.18 ± 10.39 years; *p* <0.001) than adults who remained in the study and more adults with low educational level (58.4 % vs. 41.6 %) dropped out of the study (*p* = 0.004) compared to adults with high educational level. No differences were found for Body Mass Index (BMI) (*p* = 0.204) and sex (*p* = 0.918). Both at baseline and follow-up, participants provided written informed consent. The study protocol was approved by the Ghent University Hospital Ethics Committee.

### Measures

The questionnaire, specifically developed for adults, assessed total sitting time, context-specific sitting time and its potential intrapersonal, social-cognitive and physical environmental predictors. The questionnaire showed moderate-to-good validity (ρ = 0.49) compared to activPAL data and moderate-to-good test-retest reliability (Intraclass Correlation Coefficients (ICC) = 0.77) for total sitting time on an average day [21]. Test-retest reliability for the included context-specific sitting times (i.e. TV viewing, computer use, motorized transport during leisure time, commuting and occupation-related motorized transport, and occupational sitting) showed acceptable results [21]. Furthermore, the included potential predictors of context-specific sitting time had moderate-to-excellent reliability (Kappa coefficients, ICC ≥0.41 or acceptable percentage agreement) [21]. Still, some social-cognitive variables were excluded from analyses in the present study as they revealed poor reliability (see Table 1).

**Table 1:** Overview of the included item-specific social-cognitive variables

	Item questionnaire	Baseline (mean ± SD)	Follow-up (mean ± SD)	Change-score follow-up - baseline (mean ± SD)
TV viewing				
Attitude 1^(a)^	I think watching TV is pleasant	4.08 ± 0.88	4.03 ± 0.86	−0.08 ± 0.74
Attitude 2^(a)^	Watching TV takes time away from doing other important things	2.69 ± 1.24	2.60 ± 1.16	0.04 ± 1.25
Attitude 3^(a)^	I enjoy watching TV for many hours at a time	2.68 ± 1.21	2.69 ± 1.20	0.02 ± 1.14
Attitude 4^(a)^	Watching TV is my way to relax after a school day/workday	3.48 ± 1.21	3.33 ± 1.29	−0.18 ± 1.03
Self-efficacy 1^(a)^	I consider it possible to reduce my TV time^(d)^	/	/	/
Self-efficacy 2^(a)^	I consider it possible to turn off the TV during weekend days until 5:00 p.m.	4.20 ± 1.19	4.23 ± 1.13	−0.03 ± 1.31
Self-efficacy 3^(a)^	I consider it possible to turn off the TV during meals	4.37 ± 1.07	4.46 ± 0.96	0.03 ± 1.11
Norm^(a)^	I think that I spend too much time watching TV	2.20 ± 1.16	2.16 ± 1.13	0.05 ± 1.06
Social norm^(a)^	My family members think I spend too much time watching TV	1.76 ± 0.97	1.75 ± 0.84	−0.03 ± 0.82
Social support 1^(a)^	My family members encourage me to watch less TV	1.84 ± 0.97	1.81 ± 0.88	0.04 ± 0.91
Social support 2^(a)^	My friends encourage me to watch less TV^(*f)*^	/	/	/
Modelling 1^(b)^	How long, on average, does your partner spend watching TV?	5.52 ± 2.27	5.28 ± 2.32	−0.03 ± 1.27
PC-use				
Attitude 1^(a)^	I think using a computer is pleasant in leisure time	3.68 ± 1.09	3.63 ± 1.17	−0.03 ± 1.05
Attitude 2^(a)^	Using a computer takes time away from doing other important things	2.54 ± 1.25	2.51 ± 1.23	0.04 ± 1.30
Attitude 3^(a)^	I enjoy using a computer for many hours at a time	2.45 ± 1.16	2.45 ± 1.21	0.02 ± 1.17
Attitude 4^(a)^	Using a computer is my way to relax after a school day/workday	2.47 ± 1.25	2.45 ± 1.26	0.01 ± 1.17
Self-efficacy 1^(a)^	I consider it possible that I do not use a computer for some days in the week (leisure time)	3.48 ± 1.38	3.43 ± 1.38	−0.07 ± 1.52
Self-efficacy 2^(a)^	I consider it possible to reduce my computer time in leisure time	3.16 ± 1.21	3.18 ± 1.16	−0.03 ± 1.35
Norm^(a)^	I think that I spend too much time using a computer	1.99 ± 1.13	2.03 ± 1.11	0.02 ± 1.01
Social norm ^(a)^	My family members think I spend too much time using a computer^(d)^	/	/	/
Social support 1^(a)^	My family members encourage me to spend less time using a computer in leisure time^(d)^	/	/	/
Social support 2^(a)^	My friends encourage me to spend less time using a computer in leisure time	1.80 ± 0.95	1.83 ± 0.91	0.06 ± 0.92
Modelling 1^(b)^	How long, on average, does your partner sit when using the computer (tablet, internet on smartphone, laptop, desktop,…) in leisure time?^(d)^	/	/	/
Motorized transport				
Attitude 1^(a)^	I feel lazy arriving at my destination after motorized transport^(e)^	3.80 ± 1.14	3.70 ± 1.12	−0.11 ± 1.29
Attitude 2^(a)^	I think using motorized transport is pleasant	3.50 ± 1.09	3.24 ± 1.16	−0.14 ± 1.08
Attitude 3^(a)^	I think it is pleasant to work or to rest as a passenger during motorized transport	3.01 ± 1.23	2.93 ± 1.22	0.03 ± 1.44
Self-efficacy 1^(a)^	I consider it possible to get off the bus/metro spontaneously 1 stop before my destination and to walk the remaining distance	3.01 ± 1.34	3.25 ± 1.25	0.22 ± 1.30
Self-efficacy 2^(a)^	I consider it possible to park the car somewhat further spontaneously and to walk the remaining distance	3.27 ± 1.31	3.49 ± 1.20	0.16 ± 1.14
Self-efficacy 3^(a)^	I consider it possible to take the bicycle or to go by foot spontaneously even if it is possible to use a car	4.02 ± 1.14	4.16 ± 1.03	0.14 ± 1.11
Norm^(a)^	I think that I spend too much time using motorized transport	2.39 ± 1.23	2.42 ± 1.17	0.06 ± 1.12
Social norm^(a)^	My family members think I spend too much time using motorized transport	1.95 ± 1.06	1.95 ± 0.96	0.07 ± 0.81
Social support 1^(a)^	My family members encourage me to use (more often) active transport (to bicycle or to walk)	2.85 ± 1.31	2.71 ± 1.28	−0.16 ± 1.27
Social support 2^(a)^	My friends encourage me to use (more often) active transport (to bicycle or to walk)^(*f)*^	/	/	/
Modelling 1^(c)^	The most chosen transportation possibility to go to work/school from my partner	2.05 ± 0.65	2.04 ± 0.68	−0.03 ± 0.49
Modelling 2^(c)^	The most chosen transportation possibility in leisure time from my partner	2.11 ± 0.63	2.19 ± 0.70	0.09 ± 0.60
Occupation				
Attitude 1^(a)^	My attention decreases while sitting for a long time	3.11 ± 1.32	3.23 ± 1.22	0.22 ± 1.77
Attitude 2^(a)^	I think it is pleasant to sit for a long time during working hours	2.27 ± 1.02	2.26 ± 1.01	0.03 ± 1.55
Attitude 3^(a)^	Sitting at work is an ideal opportunity to rest	2.48 ± 1.22	2.34 ± 1.25	−0.14 ± 1.68
Self-efficacy 1^(a)^	I consider it possible to do certain tasks (calling,…) while standing	3.64 ± 1.23	3.71 ± 1.14	0.18 ± 1.75
Self-efficacy 2^(a)^	I consider it possible to stand up for a while after a period of uninterrupted sitting	4.38 ± 0.85	4.23 ± 0.92	−0.15 ± 1.32
Self-efficacy 3^(a)^	I consider it possible to stand up more often during breaks	3.43 ± 1.39	3.58 ± 1.39	0.26 ± 2.12
Norm^(a)^	I think that I spend too much time sitting at work	2.54 ± 1.35	2.64 ± 1.37	0.10 ± 2.03
Social norm^(a)^	My colleagues think I spend too much time sitting	1.83 ± 0.89	1.88 ± 0.98	0.08 ± 1.33
Social support 1^(a)^	My colleagues encourage me to sit less^(d)^	/	/	/

Abbreviations: *B* baseline, *FU* follow-up, *PC-use* computer use

*Answering categories* ^(a)^: strongly disagree; somewhat disagree; neutral; somewhat agree; strongly agree

*Answering categories* ^(b)^: No TV-viewing or PC-use; only a few times a week; less than 30 min/day; 30–60 min/day; 1–2 h/day; 2–3 h/day; 3–4 h/day; more than 4 h/day

*Answering categories* ^(c)^: motorized transport; active transport (e.g. walking, bicycling)

(^d^) indicates an item that is not included due to low test-retest reliability

(^e^) indicates an item that was recoded because of negative scoring

(^f^) indicates an item that was removed from analyses due to multicollinearity in step 1

#### Sitting time

Context-specific sitting time (11 items) was assessed by targeting sitting behaviour in the past 7 days during engagement in sitting during TV viewing and computer use (both reported separately for weekday and weekend day), sitting during motorized transport during leisure time (reported separately for weekday and weekend day), sitting during motorized transport during commuting and sitting during occupation-related motorized transport (reported on an average working day), and occupation (reported on an average working day). These contexts of sitting time were measured identically at baseline and follow-up. The following answer categories were used for TV viewing and computer use: ‘none’, ‘1–15 min/day’, ‘15–30 min/day’, ‘30–60 min/day’, ‘1–2 h/day’, ‘2–3 h/day’, ‘3–4 h/day’, ‘4–5 h/day’, ‘5–6 h/day’, ‘6–7 h/day’ or ‘more than 7 h/day’. Slightly different answer categories were used for questions concerning motorized transport (‘none’, ‘1–15 min/day’, ‘15–30 min/day’, ‘30–45 min/day’, ‘45–60 min/day’, ‘60–90 min/day’, ‘90–120 min/day’, ‘2–2.5 h/day’, ‘2.5–3 h/day’, ‘3–4 h/day’, ‘4–5 h/day’, ‘5–6 h/day’, ‘6–7 h/day’ or ‘more than 7 h/day’) and occupational sitting (‘less than 2 h/day’, ‘2–3 h/day’, ‘3–4 h/day’, ‘4–5 h/day’, ‘5–6 h/day’, ‘6–7 h/day’, ‘7–8 h/day’, ‘more than 8 h/day’).

#### Correlates of context-specific sitting time

Intrapersonal variables that were assessed at baseline and follow-up were: BMI, occupational status, residential area, depressive symptoms [22], children living at home, family situation, occupational classification, educational level and sex (questions and answer categories are displayed in Table 2). Next, for each context of sitting time the following context- and item-specific social-cognitive variables were determined: attitude, self-efficacy, norm, social norm, social support and modelling (e.g. attitude 1 TV viewing: ‘I think watching TV is pleasant’; attitude 2 TV viewing: ‘watching TV takes time away from doing other important things’). The social-cognitive variables were measured both at baseline and follow-up (Table 1). The answer categories of the variables measuring attitude, self-efficacy, (social) norm and social support ranged from ‘strongly disagree’ to ‘strongly agree’. The answer categories of modelling for TV viewing and computer use ranged from ‘never’ to ‘more than 4 h/day’, and for motorized transport a dichotomous answer category was used (i.e. active transport or motorized transport).

**Table 2:** Overview of the included (changes in) intrapersonal variables

	Questionnaire item	Original answer category	Recoded variables for cross-sectional analyses (baseline)	Longitudinal analyses	
				Recoding (% or mean ± SD)	New variables based on recoding
Family situation	How would you describe your family situation?	B: 1 = single; 2 = having a partner, but living independently; 3 = living with a partner; 4 = married; 5 = widow/widower	0 [not living with a partner (=1/2/5) = 20.9 %]; 1 [living with a partner (=3/4) = 79.1 %]	-	-
Occupational classification	What is your occupational classification?	B: 1 = white collars; 2 = blue collars; 3 = others, i.e. household or no-paid job	**Dummy1:** 0 [others and white collars = 80.7 %]; 1 [blue collars = 19.3 %]	-	-
			**Dummy2:** 0 [others and blue collars = 26.7 %]; 1 [white collars = 73.3 %]		
Educational level	What is your highest achieved diploma or certificate?	B: 1 = primary school; 2 = secondary education; 3 = higher education, non-university; 4 = university	0 [low educational level (=1/2) = 47.8 %]; 1 [high educational level (=3/4) = 52.2 %]	-	-
Sex	What is your sex?	B: 1 = male; 2 = female	0 [female]; 1 [male]	-	-
Occupational status	At the moment I have/work/am/do …	B: 1 = full-time job; 2 = part-time job; 3 = household; 4 = unemployed/job-applicant; 5 = career interruption; 6 = retired; 7 = student	0 [not working (=3-7) = 11.0 %]; 1 [working (=1-2) = 89.0 %]	1-2 → 1–2 OR 3–7 → 3–7 (stable = 93.5 %); 1–2 → 3–7 (stop working = 4.9 %); 3–7 → 1–2 (start working = 1.6 %)	0 = stable 1 = stop working 2 = start working
		FU: 1 = full-time job; 2 = part-time job; 3 = household; 4 = unemployed/job-applicant; 5 = career interruption; 6 = retired; 7 = student			
Residential area	In which type of area do you live?	B: 1 = countryside; 2 = village or town; 3 = cities suburbs; 4 = city	0 [countryside and village/town = 31.5 %]; 1 [cities suburbs and city = 68.5 %]	1 → 1 OR 2 → 2 OR 3 → 3 OR 4 → 4 (stable = 94.4 %); 1–2 → 3–4 (increase = 1.1 %); 3–4 → 1–2 (decrease = 4.5 %)	0 = stable 1 = increase 2= decrease
		FU: 1 = countryside; 2 = village or town; 3 = cities suburbs; 4 = city			
Depressive symptoms	a) During the past month, have you often been bothered by feeling down, depressed, or hopeless? b) During the past month, have you often been bothered by little interest or pleasure in doing things?	a) B: 1 = yes; 2 = no b) B: 1 = yes; 2 = no	0 [no depressive symptoms (=answered “no” on both questions) = 79.6 %]; 1 [depressive symptoms (=answered “yes” on at least one of the questions) = 20.4 %]	B: Positive test (answered “yes” on at least one of the questions = 20.4 %); negative test (answered “no” on both questions = 79.6 %)	0 = stable 1 = developing depressive symptoms 2 = disappearance of depressive symptoms
		a) FU: 1 = yes; 2 = no b) FU: 1 = yes; 2 = no		FU: Positive test (answered “yes” on at least one of the questions = 20.0 %); negative test (answered “no” on both questions = 80.0 %)	
Children living at home	How many of your children still live at home?	B: … child(ren) FU: … child(ren)	0 [no child(ren) at home = 44.6 %]; 1 [yes, child(ren) at home = 55.4 %]	B: not having a child (28.4 %); not having a child still living at home (16.2 %); having child(ren) living at home (55.4 %)	0 = stable 1 = getting child(ren) 2 = no children living at home
				FU: not having a child (23.9 %); not having a child still living at home (21.7 %); having child(ren) living at home (54.3 %)	
Body Mass Index (BMI)	Self-reported height and weight	B: … cm/… kg	… kg/m^2^	/	follow-up BMI minus baseline BMI
		FU: … cm/… kg			

Abbreviations: *B* baseline, *FU* follow-up

“/” indicates that no recoding was performed. “-” indicates variables that were not included in longitudinal analyses

Furthermore, physical environmental variables specifically for each context of sitting time were included both at baseline and follow-up (see Table 3). For TV-viewing, the variable ‘TV set’ was calculated by summing the number of pay televisions and non-pay televisions available in the household and the variable ‘other TV-viewing equipment’ by summing the number of laptops, desktops, smartphones and tablets available in the household. For computer use, the variable ‘computer equipment’ was calculated by summing the number of laptops and desktops available in the household. The variable ‘other equipment for computer use’ was obtained by summing the number of smartphones, tablets, and (portable) gaming consoles available in the household. The answer categories for both equipment regarding TV-viewing and computer use (i.e. electronic devices belonging to the variables ‘TV set’, ‘other TV-viewing equipment’, ‘computer equipment’ and ‘other equipment for computer use’) ranged from ‘none’, ‘1’, ‘2’, ‘3’, ‘4’, ‘5’, to ‘more than 5’. Importantly, participants were asked to only take into account equipment regarding TV-viewing and computer use that they operate themselves. For motorized transport, participants recorded the number of operational motorized vehicles present in the household, even if they did not use any of them (e.g. cars, motorbike, moped,…). Regarding the occupational environment, participants indicated if they had standing desks at work (yes/no question) [21].

**Table 3:** Overview of the included physical environmental variables

	Items questionnaire	Baseline (mean ± SD)	Follow-up (mean ± SD)	Change-score follow-up - baseline (mean ± SD)
TV set	How many of the following electronic devices do you use and are present at your home?	1.42 ± 0.82	1.51 ± 0.84	0.04 ± 0.69
	a) pay TV^(1)^ b) no pay TV^(1)^			
Other TV-viewing equipment	How many of the following electronic devices do you use and are present at your home?	2.77 ± 1.79	3.13 ± 2.01	0.45 ± 1.68
	a) laptops^(1)^ b) desktops^(1)^ c) smartphones^(1)^ d) tablets^(1)^			
PC equipment	How many of the following electronic devices do you use and are present at your home?	1.89 ± 1.05	1.84 ± 1.04	−0.04 ± 0.96
	a) laptops^(1)^ b) desktops^(1)^			
Other equipment for computer use	How many of the following electronic devices do you use and are present at your home?	1.60 ± 1.86	1.97 ± 2.23	0.56 ± 1.68
	a) smartphones^(1)^ b) tablets^(1)^ c) gaming consoles for TV^(1)^ d) portable gaming consoles^(1)^			
Standing desks at occupation	There are standing desks (heightened desks at which one can work while standing)?^(2)^	0.13 ± 0.34	0.13 ± 0.34	/
Motorized vehicles	How many operational motorized vehicles are there present in the household, even the ones you do not use yourself?^(3)^	1.80 ± 1.16	1.77 ± 1.00	−0.14 ± 0.69

PC computer, TV television

“/” indicates that no change-score was measured

Answering categories^(1)^: ‘none’, ‘1’, ‘2’, ‘3’, ‘4’, ‘5’ or ‘more than 5’

Answering categories^(2)^: ‘yes’ or ‘no’

Answering categories^(3)^: open-ended question

#### Potential covariate of context-specific sitting time

Total PA (minutes walking, moderate-intensity PA and vigorous-intensity PA per day) was measured at baseline and follow-up using the short-IPAQ (International Physical Activity Questionnaire – last 7 day recall format), which was shown to have acceptable measurement properties [23].

### Data reduction

#### Sitting time

Context-specific sitting time was measured by determining the midpoint values of the above-mentioned answer categories. Afterwards, self-reported context-specific sitting time was calculated for an average day regarding TV viewing, computer use and motorized transport during leisure time, using the following formula: ((sitting time on a weekday * 5) + (sitting time on a weekend day * 2))/7. Total amount of motorized transport was measured by summing leisure time, commuting and occupation-related motorized transport on an average workday. Consequently, all included context-specific sitting times were calculated for an average day. A change-score for each context of sitting time was calculated by subtracting baseline measurements from follow-up measurements (i.e. follow-up minus baseline).

#### Correlates of context-specific sitting time

The baseline measurements (i.e. cross-sectional) were used to identify potential correlates of context-specific sitting time. Furthermore, changes in intrapersonal, social-cognitive and physical environmental variables from baseline to follow-up were included as potential predictors of changes in context-specific sitting times (i.e. longitudinal). Change-scores were calculated by subtracting baseline measurements from follow-up measurements (i.e. follow-up minus baseline). Intrapersonal variables that were included as potential correlates (baseline measurements) were: BMI (kg/m^2^), occupational status, residential area, depressive symptoms, children living at home, family situation, occupational classification, educational level and sex. For all above-mentioned variables, except for family situation, occupational classification, educational level and sex, a change-score was calculated. Change in family situation and occupational classification were not included in the longitudinal analyses, due to a limited variance during 1 year of follow-up. An overview of the included (change-scores of) intrapersonal variables (including scoring methods and descriptive statistics [% or mean ± SD or median]) is shown in Table 2. Furthermore, a change-score for each social-cognitive and physical environmental variable was calculated (i.e. follow-up item-specific mean minus baseline item-specific mean). All items were scored/recoded in the same direction to facilitate interpretation of the results (highest score is most positive answer). Detailed information (including scoring methods and descriptive statistics [mean ± SD]) of the social-cognitive variables can be found in Table 1. The physical environmental variable of occupational sitting was calculated based on the change in the presence of standing desks from baseline to follow-up (i.e. ‘stable’, ‘getting some’ and ‘not anymore’).

#### Potential covariate of context-specific sitting time

PA data gathered through the short-IPAQ were processed using the IPAQ scoring protocol [24]. Baseline measurement of total PA was included as a covariate in the cross-sectional analyses and a change-score for total PA (i.e. follow-up minus baseline) was included as a covariate in the longitudinal analyses.

### Statistical analyses

All analyses were conducted with SAS version 9.4 software (SAS institute Inc., Cary, NC, USA). Statistical significance was determined at α = 0.05.

#### Potential correlates of context-specific sitting time at baseline (aim a)

For the cross-sectional analyses, generalized linear regression analyses were performed using PROC GENMOD [25]. Since the dependent variables were non-normally distributed (i.e. they were positively skewed) a generalized linear model with gamma variance and log link function was used. This model yielded the best fit based on Akaike’s Information Criterion (AIC). The estimation procedure used was maximum likelihood estimation. Correlates of context-specific sitting time (i.e. TV-viewing, computer use, motorized transport and occupation) were identified using a four steps procedure, separately for each included context-specific sitting time. First, multicollinearity between the correlates was tested per level (intrapersonal, social-cognitive and physical environmental variables). When two correlates belonging to one level were strongly correlated (Pearson *r* >0.60) [26], the correlate that correlated the weakest with the context-specific sitting time (dependent variable) was removed from the specific analyses. Following this procedure, ‘my friends encourage me to watch less TV’ (social support 2 TV viewing), ‘using a computer is my way to relax after a school day/workday’ (attitude 4 computer use), ‘I consider it possible to park the car somewhat further spontaneously and to walk the remaining distance’ (self-efficacy 2 motorized transport) and ‘my family members encourage me to use (more often) active transport’ (social support 1 motorized transport) were removed. Secondly, for each level, conducted model was fitted containing all correlates within that level (i.e. three regression models per context-specific sitting time). The correlates that showed levels of significance *p* <0.10 with the dependent variable were included in the next step [27, 28]. In step 3, multicollinearity between the correlates that yielded a *p* <0.10 in step 2 was tested with Pearson correlation coefficients. None of these correlates were highly correlated (*r* >0.60) with each other. In step 4, the correlates with *p* <0.10 in step 2 were combined into one model. The results of step 4 were presented and discussed. All the analyses were adjusted for total PA at baseline.

#### Changes in potential predictors related to changes in context-specific sitting time (aim b)

For the longitudinal analyses, the change-scores for the context-specific sitting times were normally distributed and linear regression analyses were performed using PROC CALIS [29]. The estimation procedure used was full-information maximum likelihood. Full-information maximum likelihood effectively utilizes all available information and is the recommended estimation procedure when dealing with missing data on both outcome and predictor variables, as was the case using the present longitudinal data [29]. The analyses examined if change-scores of potential predictors predicted changes in context-specific sitting time. Analyses were performed using the same stepwise approaches used to identify the correlates (as described above). In step 1, ‘my friends encourage me to use (more often) active transport’ (social support 2 motorized transport) and ‘my friends encourage me to watch less TV’ (social support 2 TV viewing) were removed due to multicollinearity. Secondly, for each level, multiple regression models were conducted containing all change-scores of potential predictors within that level (i.e. three regression models per context-specific sitting time). In step 3, none of remaining change-scores of potential predictors were highly correlated with each other. In step 4, the change-scores of potential predictors with *p* <0.10 in step 2 were combined into one multiple linear regression model. The results of step 4 were presented and discussed. All the analyses were adjusted for change in total PA between baseline and follow-up and baseline context-specific sitting time. Furthermore the longitudinal analyses were adjusted for age and educational level at baseline, based on information obtained from the drop-out analyses.

## Results

### Sample characteristics

The socio-demographic characteristics, BMI and context-specific sitting times at baseline and follow-up can be found in Table 4.

**Table 4:** Sample characteristics at baseline and follow-up

	Baseline	Follow-up
Age (years, mean (SD))	43.3 (10.6)	46.0 (10.4)
Male gender (%)	45.5	45.2
High educational level (%)	52.2	59.4
BMI (kg/m^2^, mean (SD))	24.6 (3.5)	24.4 (3.4)
Not having depressive symptoms (%)	79.6	80.0
Family situation		
Married or living with partner (%)	79.0	80.5
Widow/widower (%)	1.7	1.6
Single (%)	12.2	11.4
Partner, but living apart (%)	7.1	6.5
Occupational status		
Full-time job (%)	71.9	72.4
Part-time job (%)	17.1	18.9
Household (%)	5.4	3.2
Unemployed/job-applicant (%)	2.7	1.1
Career interruption (%)	1.0	2.7
Retirement (%)	1.0	1.6
Student (%)	1.0	0.0
Occupational classification		
White collar (%)	71.9	73.0
Blue collar (%)	23.4	24.6
Another job (e.g. household) (%)	4.7	2.4
TV viewing time (min/average day, mean (SD))	129.0 (74.6)	122.0 (75.9)
Computer use (min/average day, mean (SD))	58.5 (69.0)	54.6 (61.0)
Motorized transport (min/average day, mean (SD))	75.6 (76.2)	69.5 (60.1)
Occupational sitting time (min/average day, mean (SD))	216.4 (170.2)	222.0 (158.5)

The participants who remained in the study differed in certain characteristics compared to the population of Flemish adults (2012–2013), as people living in Flanders had a mean BMI of 25.3 kg/m^2^ (vs 24.4 kg/m^2^), 34.0 % had a part-time job (vs 18.9 %), 24.6 % was lowly-educated (vs 40.6 %), and 12.5 % showed to have symptoms of a depressive disorder (vs 20.0 %) [30–32].

### Aim a: identification of intrapersonal, social-cognitive and physical environmental correlates of TV-viewing, computer use, motorized transport and occupational sitting at baseline

An overview of the item-specific correlates, associated with TV time, computer use, motorized transport and occupation at baseline, is reported in Table 5. Four variables were significantly related to *sitting while watching TV*. Having a high educational level was associated with 23 % less sitting while watching TV compared to having a low educational level. Furthermore, a one-unit higher score for ‘I enjoy watching TV for many hours’ (attitude 3) and ‘I find TV a way to relax’ (attitude 4) was associated with respectively 19 and 12 % more sitting while watching TV. Also, a one-unit higher score for ‘time partner spend watching TV’ (modelling 1) was associated with 5 % more sitting while watching TV.

**Table 5:** Item-specific correlates of sitting during TV time, computer use, motorized transport and occupation (cross-sectional analyses for baseline data)

Correlates	Dependent variables											
	Sitting while watching TV			Sitting during PC use			Sitting during motorized transport			Occupational sitting		
	Estimate	95 % CI	*p*	Estimate	95 % CI	*p*	Estimate	95 % CI	*p*	Estimate	95 % CI	*p*
Intrapersonal variables												
Occupational status	-	-	-	-	-	-	-	-	-	x	x	x
Residential area	-	-	-	-	-	-	-	-	-	-	-	-
Depressive symptoms	-	-	-	-	-	-	-	-	-	-	-	-
Children living at home	-	-	-	0.79	0.64;0.97	*	1.31	1.08;1.59	**	-	-	-
Body Mass Index	-	-	-	-	-	-	-	-	-	-	-	-
Family situation	-	-	-	0.73	0.56;0.97	*	-	-	-	-	-	-
Occupational classification^												
*Blue collars*	-	-	-	1.09	0.57;2.07	ns	-	-	-	2.50	1.35;4.66	**
*White collars*	-	-	-	1.24	0.68;2.26	ns	-	-	-	3.36	1.86;6.05	***
Educational level	0.77	0.68;0.88	***	-	-	-	-	-	-	1.32	1.03;1.70	*
Sex	-	-	-	1.01	0.82;1.26	ns	1.83	1.50;2.23	***	-	-	-
Social-cognitive variables												
Attitude 1	-	-	-	1.34	1.18;1.52	***	-	-	-	-	-	-
Attitude 2	-	-	-	-	-	-	-	-	-	-	-	-
Attitude 3	1.19	1.12;1.27	***	1.17	1.05;1.31	**	-	-	-	-	-	-
Attitude 4	1.12	1.06;1.19	***	x	x	x	x	x	x	x	x	x
Self-efficacy 1	x	x	x	0.87	0.81;0.95	**	-	-	-	-	-	-
Self-efficacy 2	-	-	-	-	-	-	x	x	x	-	-	-
Self-efficacy 3	-	-	-	x	x	x	0.81	0.75;0.89	***	-	-	-
Norm	1.06	0.99;1.12	ns	1.24	1.12;1.37	***	1.14	1.05;1.24	**	-	-	-
Social norm	-	-	-	x	x	x	-	-	-	-	-	-
Social support 1	-	-	-	x	x	x	x	x	x	x	x	x
Social support 2	x	x	x	-	-	-	-	-	-	x	x	x
Modelling 1	1.05	1.03;1.08	***	x	x	x	-	-	-	x	x	x
Modelling 2	x	x	x	x	x	x	-	-	-	x	x	x
Physical environment												
TV set	1.07	0.98;1.18	ns	x	x	x	x	x	x	x	x	x
Other TV-viewing equipment	0.98	0.94;1.02	ns	x	x	x	x	x	x	x	x	x
PC equipment (desktop & laptop)	x	x	x	1.08	0.96;1.21	ns	x	x	x	x	x	x
Other equipment for computer use	x	x	x	-	-	-	x	x	x	x	x	x
Standing desks at occupation	x	x	x	X	x	x	x	x	x	-	-	-
Motorized vehicles	x	x	x	X	x	x	1.07	0.98;1.17	ns	x	x	x

Potential correlates were identified by using baseline measurements. “x” indicates correlates not inserted in analyses for context-specific sedentary behaviour. “-” indicates correlates that showed levels of significance *p* ≥.10 at the second step. For occupational classification (^), ‘others, i.e. household or no-paid job’ was used as the reference category

Occupational status was not inserted in the analyses regarding occupation, due to model fit. The scoring of occupational classification (dummmy1 and dummy2) can be found in Table 2. All analyses were adjusted for total physical activity

Abbreviations: *PC* computer, *TV* television, *ns* not significant, *CI* confidence interval. Correlates inserted in the fourth step were labelled: ***p <.001; **p <.01; *p <.05

Six variables were significantly related to *sitting while using a computer*. Still having child(ren) living at home, was associated with 21 % less sitting while using a computer compared to not having children living at home. Being married or living together with a partner was associated with 27 % less sitting while using a computer compared to living independently. A one-unit higher score for ‘I think using a computer is pleasant’ (attitude 1), ‘I enjoy using a computer for many hours’ (attitude 3) and ‘I think that I spend too much time on the computer’ (norm) was associated with respectively 34, 17 and 24 % more sitting while using a computer. A one-unit higher score for ‘I consider it possible that I do not use a computer for some days in the week’ (self-efficacy 1) was associated with 13 % less sitting while using a computer.

Three variables were significantly related to *sitting during motorized transport*. Being male was associated with 83 % more motorized transport compared to being female. A one-unit higher score for ‘I think that I spend too much time using motorized transport’ (norm) was associated with 14 % more sitting during motorized transport. A one-unit higher score for ‘I consider it possible to take the bicycle or to go by foot spontaneously even if it is possible to use a car’ (self-efficacy 3) was associated with 19 % less sitting during motorized transport.

Three variables were significantly related to *occupational sitting*. Having a blue or white collar job was associated with more occupational sitting (2.5 and 3.36 times higher, respectively) compared to having no paid job (e.g. household). Having a white collar job was associated with 34 % higher occupational sitting time compared to having a blue collar job. Having a high educational level was associated with 32 % higher occupational sitting compared to having a low educational level.

### Aim b: relationship between changes in potential predictors (i.e. intrapersonal, social-cognitive and physical environmental) from baseline to follow-up and changes in TV-viewing, computer use, motorized transport and occupational sitting

An overview of the item-specific change-scores (predictors), associated with change in sitting while watching TV, computer use, motorized transport and occupational sitting over a 1-year follow-up period, is reported in Table 6. Three change-scores were significantly related to *change in TV viewing*. No changes in the variable ‘children living at home’ at follow-up was associated with 22.58 min/day less TV viewing at follow-up compared to being in the reference group (i.e. having no children). An increase from baseline to follow-up with one unit on the five-point Likert scale for ‘I enjoy watching TV for many hours at a time’ (attitude 3) was associated with 7.96 min/day more sitting while watching TV at follow-up. An increase from baseline to follow-up with one unit on the eight-point Likert scale for ‘time partner spend watching TV’ (modelling 1) was associated with 9.91 min/day more sitting while watching TV at follow-up.

**Table 6:** Item-specific change-scores (predictors) of sitting during TV time, computer use, motorized transport and occupation (longitudinal analyses)

Predictors	Dependent variables							
	Sitting while watching TV		Sitting during PC use		Sitting during motorized transport		Occupational sitting	
	B (SE)	*p*	B (SE)	*p*	B (SE)	*p*	B (SE)	*p*
Intrapersonal variables								
Body Mass Index	-	-	−2.95 (1.72)	ns	-	-	-	-
Occupational status^								
*Stop working (from BL to FU)*	-	-	-	-	−37.65 (11.86)	**	−98.22 (30.54)	**
*Start working (from BL to FU)*	-	-	-	-	−46.80 (20.13)	*	−27.85 (52.06)	ns
Residential area^								
*Increase (from BL to FU)*	-	-	-	-	-	-	82.58 (63.87)	ns
*Decrease (from BL to FU)*	-	-	-	-	-	-	−79.40 (32.56)	*
Depressive symptoms								
*Developing depressive symptoms (from BL to FU)*	-	-	-	-	-	-	-	-
*Disappearance of depressive symptoms (from BL to FU)*	-	-	-	-	-	-	-	-
Children living at home^								
*Stable (from BL to FU)*	−22.58 (9.84)	*	-	-	-	-	-	-
*Getting children (from BL to FU)*	3.81 (25.66)	ns	-	-	-	-	-	-
*No children living at home (from BL to FU)*	−18.21 (20.81)	ns	-	-	-	-	-	-
Social-cognitive variables								
Attitude 1	-	-	-	-	-	-	-	-
Attitude 2	-	-	-	-	-	-	-	-
Attitude 3	7.96 (3.40)	*	-	-	-	-	-	-
Attitude 4	-	-	-	-	x	x	x	x
Self-efficacy 1	x	x	-	-	−4.59 (2.36)	ns	-	-
Self-efficacy 2	-	-	-	-	8.48 (2.70)	**	-	-
Self-efficacy 3	-	-	x	x	-	-	-	-
Norm	-	-	-	-	-	-	-	-
Social norm	9.76 (5.00)	ns	x	x	-	-	-	-
Social support 1	-	-	x	x	-	-	x	x
Social support 2	-	-	-	-	-	-	x	x
Modelling 1	9.91 (2.93)	***	x	x	16.47 (5.43)	**	x	x
Modelling 2	x	x	x	x	-	-	x	x
Physical environment								
TV set	-	-	x	x	x	x	x	x
Other TV-viewing equipment	−2.19 (2.14)	ns	x	x	x	x	x	x
PC equipment (desktop & laptop)	x	x	-	-	x	x	x	x
Other equipment for computer use	x	x	-	-	x	x	x	x
Standing desks at occupation								
*Yes, getting some (from BL to FU)*	x	x	x	x	x	x	-	-
*No, not anymore (from BL to FU)*	x	x	x	x	x	x	-	-
Motorized vehicles	x	x	x	x	10.73 (3.68)	**	x	x

“x” indicates predictors not inserted in analyses for context-specific sedentary behaviour. “-” indicates predictors that showed levels of significance *p* ≥.10 at the second step. All analyses were adjusted for baseline context-specific sedentary behaviour, age, educational level and change-score for total physical activity

Abbreviations: *BL* baseline, *FU* follow-up, *PC* computer, *TV* television, *SE* standard error, *ns* not significant. Predictors inserted in the fourth step were labelled: ***p <.001; **p <.01; *p <.05. B-values can be interpreted as change in minutes/day of context-specific sitting time. The reference category (^) for occupational status and residential area was ‘being in the stable group’; for children living at home, ‘having no children’ was used as the reference category

Five change-scores were significantly related to *change in sitting during motorized transport*. Stop and start working at follow-up was associated with 37.65 and 46.80 min/day less motorized transport at follow-up compared to being in the reference group (i.e. being in the stable group), respectively. An increase from baseline to follow-up with one unit on the five-point Likert scale for ‘I consider it possible to park the car somewhat further spontaneously and to walk the remaining distance’ (self-efficacy 2) was associated with 8.48 min/day more sitting during motorized transport at follow-up. More active transport to go to work/school (modelling 1) from baseline to follow-up of the partner was associated with 16.47 min/day more sitting during motorized transport at follow-up of the respondent. An increase from baseline to follow-up in the number of motorized vehicles was associated with 10.73 min/day more sitting during motorized transport at follow-up.

Two change-scores were significantly related to *change in occupational sitting*. Stop working at follow-up was associated with 98.22 min/day less occupational sitting at follow-up compared to being in the stable group. Moving to a smaller residential area at follow-up was associated with 79.40 min/day less occupational sitting at follow-up compared to being in the stable group.

The results showed no significant relations to *change in sitting during computer use*.

## Discussion

The present study investigated cross-sectional and longitudinal relationships of intrapersonal, social-cognitive and physical environmental variables with highly prevalent context-specific sitting times (i.e. TV viewing, computer use, motorized transport and occupation) in adults [7].

The cross-sectional analyses revealed different correlates for sitting while watching TV, during computer use, during motorized transport and occupational sitting. Social-cognitive correlates were most frequently related to context-specific sitting time, followed by intrapersonal correlates. No correlates at the physical environmental level were identified. Overall, attitude, self-efficacy and (social) norm were most consistently related to context-specific sitting time, especially for the SRSBs. For TV viewing, the enjoyment that adults experience while watching TV for many hours and the aspect that TV viewing is their way to relax after work/school were positively associated with TV time. In line with the present finding, enjoyment was a correlate of high levels of TV viewing in Australian adults [33]. Furthermore, modelling of the partner appeared to be positively related to TV viewing, which may be explained by the fact that partners have some similar interests (e.g. programs on the TV) and/or that watching TV is a family/social moment. In line with the results for TV viewing, attitude (i.e. pleasure and enjoyment) appeared to be an important correlate of computer use. Rhodes et al. [17] also indicated that positive attitudes towards SRSBs were related to higher sitting times (e.g. TV viewing and computer use). Furthermore, a positive self-efficacy regarding limiting the frequency of computer use was related to lower levels of computer use. A cross-sectional Belgian study by Van Dyck et al. [34] found associations between social-cognitive variables and TV viewing and leisure-time Internet use in adulthood, of which self-efficacy and pros and cons to reduce SRSBs were the most dominant correlates. Consequently, these findings highlight the importance of the ‘Attitudes-Social influences-Efficacy’ model (ASE model), especially for the SRSBs. In the ASE model, which is based on the theory of planned behaviour [35], a health-related behaviour (e.g. sedentary behaviour) is explained by intention which in turn is determined by attitudes, social influences and self-efficacy [36–38].

Less social-cognitive correlates were found for the non-SRSBs, however, self-efficacy and norm were associated with motorized transport. In addition, intrapersonal variables were identified as correlates of context-specific sitting time. Present findings of the intrapersonal correlates were in line with previous research showing that educational level was negatively associated with TV viewing [17] and not having children (anymore) at home or being single (or living independently) were associated with high levels of computer use [10, 17]. Previous research also identified that being male was associated with higher levels of motorized transport [19] and having a white collar job or having a high educational level was associated with high levels of occupational sitting time [39, 40]. These intrapersonal, mostly non-modifiable, correlates can indicate potential high-risk sedentary subgroups.

The longitudinal analyses revealed different predictors for sitting while watching TV, sitting during motorized transport and occupational sitting. As was the case for the cross-sectional analyses, changes in social-cognitive variables, predominantly variables of the ASE model, were the most consistent predictors of changes in sitting while watching TV and sitting during motorized transport. Consequently, the ASE model may be particularly useful to develop effective interventions in the future [35]. Importantly, the social-cognitive variables were analysed item-specific (i.e. no scales), as this ensured more detailed information for setting up future interventions [34]. The different social-cognitive variables (e.g. attitude 1–4) belonging to one construct (e.g. attitude) reflected all important aspects of that associated construct. So, as the content of these variables belonging to one construct slightly differed, it was relevant to analyse the items separately in order to maintain this specific information. Furthermore, some variables are also more easily modifiable compared to other variables belonging to the same construct. Therefore, this item-specific information will support the development of effective future interventions. An increase from baseline to follow-up for enjoying watching TV for many hours and the time their partner spend watching TV predicted more TV viewing at follow-up. These findings highlight that TV viewing is strongly present in daily life of adults, and thus may be difficult to minimize in future interventions, because watching TV may be strongly habitual and adults themselves and their family (like to) watch many hours TV. However, the present results revealed the importance of the partner/family for watching TV. Therefore, future interventions should not only be aimed at the individual but also at the family level. Future interventions should attempt to replace some TV time by social and (low) physical demanding activities. Therefore, it will be important to gather information on social activities that families consider enjoyable to incorporate during leisure time. For example, a family-based walking activity is one of the possibilities, as it can be easily integrated in leisure time and it is largely free [41]. Furthermore, walking is a low PA demanding event, so that it can be introduced to people with overweight, unfit persons or people with low levels of PA [42–44]. Still, more information is warranted about the sustainability of walking time as the replacement of TV time. Besides replacing some TV time, future interventions strategies may also focus on interrupting sitting during TV time (e.g. standing up during commercials), as enjoyment was positively associated with watching TV. These kind of activity breaks may also have positive effects on health [5, 6]. For the non-SRSBs, only significant social-cognitive predictors were found for motorized transport, of which a more positive change of self-efficacy and more active transport during commuting from baseline to follow-up of the partner were associated with more sitting during motorized transport at follow-up. These unexpected findings may be explained by the fact that both occupational motorized transport and motorized transport during leisure time were included in the calculation of motorized transport. It is possible that the positive change of self-efficacy was more associated with transport during leisure time, as the use of transport for occupational purposes is not always a free choice (e.g. too long distance to work for using active transport). Further, future research should also differentiate between public (e.g. train) and non-public (e.g. car) motorized transport, as using public motorized transport has been positively associated with daily walking time [45]. At the intrapersonal level, stop and start working at follow-up was associated with less motorized transport at follow-up compared to being in the stable group. Also, a stable child status (i.e. (no) children living at home) at follow-up was associated with less TV viewing at follow-up compared to being in the reference group (i.e. having no children). Furthermore, moving to a smaller residential area at follow-up was associated with less occupational sitting time at follow-up compared to being in the stable group. These findings highlight that changes in daily life, like having a new job, can have implications for sitting time. At the physical environmental level, an increase in number of motorized vehicles over a 1-year follow-up period was associated with more motorized transport at follow-up.

An important finding of the present study is the need for family interventions to minimize context-specific sitting time, as both intrapersonal- and social-cognitive variables were associated with context-specific sitting time. Further, most of the cross-sectional correlates differed from the longitudinal predictors, highlighting that correlates may be less informative to guide future interventions to change context-specific sitting time. Only for sitting while watching TV, two correlates were also found to be associated with change in TV time in the longitudinal analyses, namely ‘I enjoy watching TV for many hours at a time’ and ‘time partner spend watching TV’. The present findings can contribute to the development of effective future interventions, as until now the evidence on predictors of (context-specific) sitting time is scarce, especially information on social-cognitive determinants is lacking [9, 17, 46]. Still, more longitudinal research is needed to confirm these findings. Furthermore, both the cross-sectional and longitudinal results showed limited evidence for associations between the physical environment and context-specific sitting time, maybe due to some ceiling effects (i.e. limited variance in the reported answers of the physical environmental variables). For example, no variation was found between baseline and follow-up answers related to the availability of standing desks at work. Consequently, more research is warranted to investigate whether actual changes in physical environmental variables (e.g. experimental study designs) may reveal variations in the reported answers and thus possibly lead to significant associations with sitting time outcomes. To date several interventions, mostly focussing on changes in the environment, such as implementing height-adjustable desks [47–49], showed decreases in adults’ occupational sitting time. Also in the review of Neuhaus et al. [50] it was concluded that the implementation of activity-permissive workstations, including treadmills or height-adjustable desks, in the work office can effectively decrease sitting time and this with no impact on work performance. It should be noted however, that in the present study, the focus was particularly on the home environment (i.e. screen-related equipment and motorized vehicles). Additional and more extensive research to determine modifiable environmental predictors is warranted (e.g. work office-layout, neighbourhood walkability and safety from crime) [2, 9]. The combination of individual and environmental (and also organizational) elements in future interventions aiming to minimize sitting time (i.e. multicomponent interventions) will be of importance, as multiple levels of predictors influence the different contexts of sitting time [9, 51].

A first limitation of the present study is the substantial drop-out at follow-up (drop-out = 36.45 %) resulting in a relatively small sample size. Furthermore, the participants who remained in the study differed in certain characteristics compared to the population of Flemish adults (see results). Consequently, more research is needed to verify the generalizability of the present findings for adults with overweight, adults with a part-time job, high-educated adults and adults with no symptoms of a depressive disorder. A second limitation is the lack of objective measurements of context-specific sitting time, however, the questionnaire chosen has been shown to have acceptable validity and test-retest reliability results [21]. A major strength of the present study was the inclusion of a range of correlates/predictors situated at different ecological levels. In addition, we studied several highly prevalent context-specific sitting times, of which both screen-related and non-screen-related behaviours were investigated. Also, the longitudinal study design had a major added value, as limited evidence is available on causal relationships.

## Conclusions

The results indicated that the cross-sectional correlates of sitting during TV viewing, computer use, motorized transport and occupation mostly differed from the longitudinal predictors of these context-specific sitting behaviours. Variables belonging to the ASE model should be targeted in future interventions, as attitude, self-efficacy, (social) norm and modelling were most consistently related to context-specific sitting. The results from the longitudinal analyses suggest that intervention developers should acknowledge the positive association between enjoyment and modelling of the partner with sitting during TV viewing. Future interventions should attempt to replace some TV time by social and (low) physical demanding activities that families consider enjoyable to incorporate during leisure time. Also, long periods of uninterrupted sitting during TV viewing may be limited by activity breaks during commercials, as enjoyment was positively associated with watching TV. For motorized transport, intervention developers may focus on important periods in life (e.g. stop and start working), as these periods may introduce new health-related choices. Furthermore, increasing adults’ self-efficacy about parking the car somewhat further in order to walk the remaining distance may somewhat minimize sitting during motorized transport during leisure time. However, additional research is required to differentiate between occupational motorized transport and motorized transport during leisure time. Importantly, the results highlighted the need for family interventions to minimize (context-specific) sitting time, as both intrapersonal- and social-cognitive variables (e.g. time partner spend watching TV) were associated with context-specific sitting time. Future interventions should take this information into account, as solely focusing on the individual can possibly be too limited to achieve meaningful changes in sitting time. The present study found limited evidence for associations between the physical environmental variables and context-specific sitting time, however, more research is needed to determine if changes in the environment are effective in minimizing context-specific sitting time.

## Abbreviations

SB: sedentary behaviour
METs: metabolic equivalents
PA: physical activity
TV: television
SRSBs: screen-related sedentary behaviours
BMI: body mass index
ICC: intraclass correlation coefficient
IPAQ: international physical activity questionnaire
SD: standard deviation
AIC: Akaike’s information criterion
ASE: attitudes-social influences-efficacy
PC: computer

## References

[1] Barnes J, Behrens TK, Benden ME, Biddle S, Bond D, Brassard P (2012). Letter to the Editor: standardized use of the terms “sedentary” and “sedentary behaviours”. Appl Physiol Nutr Metab.

[2] Owen N, Salmon J, Koohsari MJ, Turrell G, Giles-Corti B (2014). Sedentary behaviour and health: mapping environmental and social contexts to underpin chronic disease prevention. Br J Sports Med.

[3] de Rezende LF, Rodrigues Lopes M, Rey-Lopez JP, Matsudo VK, Luiz OC (2014). Sedentary behavior and health outcomes: an overview of systematic reviews. Plos One.

[4] Asztalos M, Cardon G, De Bourdeaudhuij I, DeCocker K. Cross-Sectional Associations Between Sitting Time and Several Aspects of Mental Health in Belgian Adults. J Phys Act Health. 2014. Published Online First 28 Sep 2014. 10.1123/jpah.2013-0513 25329499

[5] Healy GN, Dunstan DW, Salmon J, Cerin E, Shaw JE, Zimmet PZ (2008). Breaks in sedentary time: beneficial associations with metabolic risk. Diabetes Care.

[6] Healy GN, Winkler EA, Owen N, Anuradha S, Dunstan DW (2015). Replacing sitting time with standing or stepping: associations with cardio-metabolic risk biomarkers. Eur Heart J.

[7] Wijndaele K, DE Bourdeaudhuij I, Godino JG, Lynch BM, Griffin SJ, Westgate K (2014). Reliability and validity of a domain-specific last 7-d sedentary time questionnaire. Med Sci Sport Exer.

[8] Chastin SFM, Schwarz U, Skelton DA (2013). Development of a Consensus Taxonomy of Sedentary Behaviors (SIT): report of Delphi Round 1. Plos One.

[9] Owen N, Sugiyama T, Eakin EE, Gardiner PA, Tremblay MS, Sallis JF (2011). Adults’ sedentary behavior determinants and interventions. Am J Prev Med.

[10] Uijtdewilligen L, Twisk JW, Singh AS, Chinapaw MJ, van Mechelen W, Brown WJ (2014). Biological, socio-demographic, work and lifestyle determinants of sitting in young adult women: a prospective cohort study. Int J Behav Nutr Phy.

[11] Barnett I, van Sluijs E, Ogilvie D, Wareham NJ (2014). Changes in household, transport and recreational physical activity and television viewing time across the transition to retirement: longitudinal evidence from the EPIC-Norfolk cohort. J Epidemiol Community Health.

[12] De Cocker KA, van Uffelen JG, Brown WJ (2010). Associations between sitting time and weight in young adult Australian women. Prev Med.

[13] Ding D, Sugiyama T, Winkler E, Cerin E, Wijndaele K, Owen N (2012). Correlates of change in adults’ television viewing time: a four-year follow-up study. Med Sci Sport Exer.

[14] Ekelund U, Brage S, Besson H, Sharp S, Wareham NJ (2008). Time spent being sedentary and weight gain in healthy adults: reverse or bidirectional causality?. Am J Clin Nutr.

[15] Touvier M, Bertrais S, Charreire H, Vergnaud AC, Hercberg S, Oppert JM (2010). Changes in leisure-time physical activity and sedentary behaviour at retirement: a prospective study in middle-aged French subjects. Int J Behav Nutr Phy.

[16] Uijtdewilligen L, Singh AS, Chinapaw MJ, Twisk JW, van Mechelen W (2015). Person-related determinants of TV viewing and computer time in a cohort of young Dutch adults: who sits the most?. Scand J Med Sci Sports.

[17] Rhodes RE, Mark RS, Temmel CP (2012). Adult sedentary behavior a systematic review. Am J Prev Med.

[18] Mummery WK, Schofield GM, Steele R, Eakin EG, Brown WJ (2005). Occupational sitting time and overweight and obesity in Australian workers. Am J Prev Med.

[19] Van Dyck D, Cerin E, Conway TL, De Bourdeaudhuij I, Owen N, Kerr J (2012). Associations between perceived neighborhood environmental attributes and adults’ sedentary behavior: findings from the USA, Australia and Belgium. Soc Sci Med.

[20] Stamatakis E, Grunseit AC, Coombs N, Ding D, Chau JY, Phongsavan P (2014). Associations between socio-economic position and sedentary behaviour in a large population sample of Australian middle and older-aged adults: The Social, Economic, and Environmental Factor (SEEF) Study. Prev Med.

[21] Busschaert C, De Bourdeaudhuij I, Van Holle V, Chastin SF, Cardon G, De Cocker K (2015). Reliability and validity of three questionnaires measuring context-specific sedentary behaviour and associated correlates in adolescents, adults and older adults. Int J Behav Nutr Phy.

[22] Whooley MA, Avins AL, Miranda J, Browner WS (1997). Case-finding instruments for depression - Two questions are as good as many. J Gen Intern Med.

[23] Craig CL, Marshall AL, Sjostrom M, Bauman AE, Booth ML, Ainsworth BE (2003). International physical activity questionnaire: 12-country reliability and validity. Med Sci Sport Exer.

[24] Guidelines for Data Processing and Analysis of the International Physical Activity Questionnaire (IPAQ) – Short and Long Forms. November 2005. https://sites.google.com/site/theipaq/scoring-protocol.

[25] SAS Institute Inc (2015). SAS/STAT® 14.1 User’s Guide.

[26] De Bourdeaudhuij I, Lefevre J, Deforche B, Wijndaele K, Matton L, Philippaerts R (2005). Physical activity and psychosocial correlates in normal weight and overweight 11 to 19 year olds. Obes Res.

[27] De Bourdeaudhuij I, Sallis J (2002). Relative contribution of psychosocial variables to the explanation of physical activity in three population-based adult samples. Prev Med.

[28] Busschaert C, Cardon G, Van Cauwenberg J, Maes L, Van Damme J, Hublet A (2015). Tracking and predictors of screen time from early adolescence to early adulthood: a 10-year follow-up study. J Adolesc Health.

[29] Allison DP. Handling Missing Data by Maximum Likelihood. Statistical Horizons, Haverford, PA, USA. 2012. http://statisticalhorizons.com/wp-content/uploads/MissingDataByML.pdf.

[30] Vlaamse Arbeidsrekening (bewerking Steunpunt WSE/Departement WSE, bewerking SVR). http://www.stadsmonitor.be/sites/default/files/thumbnails/image/149-tabel.jpg.

[31] Scientific Institute of Public Health, Operational Direction Public Health and Surveillance (HISIA : Belgian Health Interview Survey – Interactive Analysis). https://hisia.wiv-isp.be/SitePages/Home.aspx.

[32] Steunpunt Werk en Sociale Economie. 2015. http://www.steunpuntwse.be/cijfers?field_collectie_tid=All&field_indicator_tid=513&items_per_page=50.

[33] Salmon J, Owen N, Crawford D, Bauman A, Sallis JF (2003). Physical activity and sedentary behavior: a population-based study of barriers, enjoyment, and preference. Health Psychol.

[34] Van Dyck D, Cardon G, Deforche B, Owen N, De Cocker K, Wijndaele K (2011). Socio-demographic, psychosocial and home-environmental attributes associated with adults’ domestic screen time. BMC Public Health.

[35] Ajzen I (1991). The theory of planned behavior. Organ Behav Hum Dec.

[36] de Vries H, Backbier E (1994). Self-efficacy as an important determinant of quitting among pregnant women who smoke: the phi-pattern. Prev Med.

[37] Devries H, Backbier E, Kok G, Dijkstra M (1995). The impact of social influences in the context of attitude, self-efficacy, intention, and previous behavior as predictors of smoking onset. J Appl Soc Psychol.

[38] De Vries H, Dijkstra M, Kuhlman P (1988). Self-efficacy: the third factor besides attitude and subjective norm as a predictor of behavioural intentions. Health Research.

[39] Vandelanotte C, Duncan MJ, Short C, Rockloff M, Ronan K, Happell B (2013). Associations between occupational indicators and total, work-based and leisure-time sitting: a cross-sectional study. BMC Public Health.

[40] Wallmann-Sperlich B, Bucksch J, Schneider S, Froboese I (2014). Socio-demographic, behavioural and cognitive correlates of work-related sitting time in German men and women. BMC Public Health.

[41] Milton K, Kelly P, Bull F, Foster C (2011). A formative evaluation of a family-based walking intervention-Furness Families Walk4Life. BMC Public Health.

[42] Davison RC, Grant S (1993). Is walking sufficient exercise for health?. Sports Med.

[43] Morris JN, Hardman AE (1997). Walking to health. Sports Med.

[44] Siegel PZ, Brackbill RM, Heath GW (1995). The epidemiology of walking for exercise: implications for promoting activity among sedentary groups. Am J Public Health.

[45] Morency C, Trepanier M, Demers M (2011). Walking to transit: an unexpected source of physical activity. Transp Policy.

[46] Neuhaus M, Healy GN, Fjeldsoe BS, Lawler S, Owen N, Dunstan DW (2014). Iterative development of Stand Up Australia: a multi-component intervention to reduce workplace sitting. Int J Behav Nutr Phy.

[47] Healy GN, Eakin EG, Lamontagne AD, Owen N, Winkler EA, Wiesner G (2013). Reducing sitting time in office workers: short-term efficacy of a multicomponent intervention. Prev Med.

[48] Alkhajah TA, Reeves MM, Eakin EG, Winkler EA, Owen N, Healy GN (2012). Sit-stand workstations: a pilot intervention to reduce office sitting time. Am J Prev Med.

[49] Kerr J, Takemoto M, Bolling K, Atkin A, Carlson J, Rosenberg D (2016). Two-arm randomized pilot intervention trial to decrease sitting time and increase sit-to-stand transitions in working and non-working older adults. Plos One.

[50] Neuhaus M, Eakin EG, Straker L, Owen N, Dunstan DW, Reid N (2014). Reducing occupational sedentary time: a systematic review and meta-analysis of evidence on activity-permissive workstations. Obes Rev.

[51] Dunstan DW, Wiesner G, Eakin EG, Neuhaus M, Owen N, LaMontagne AD (2013). Reducing office workers’ sitting time: rationale and study design for the Stand Up Victoria cluster randomized trial. BMC Public Health.

